# 5 years follow up study on changes of romanian psychiatric residents’ opinion on factors which influence their decision to emigrate

**DOI:** 10.1192/j.eurpsy.2021.474

**Published:** 2021-08-13

**Authors:** A. Mihai, S. Trandafir, L. Duica, A. Mihai, C. Lungu, C. Pirlog

**Affiliations:** 1 Me2, GE PALADE University of Medicine, Pharmacy, Science and Technology of Târgu Mure, Târgu Mureș, Romania; 2 Psychiatry, Carol Davila University of Medicine and Pharmacy, Bucharest, Romania; 3 Psychiatry, Lucian Blaga University, Sibiu, Sibiu, Romania; 4 Psychiatry, Iuliu Hatieganu University of Medicine and Pharmacy of Cluj Napoca, Cluj Napoca, Romania; 5 Statistics, Spiru Haret University of Bucharest, Bucharest, Romania; 6 Sociology, University of Medicine and Pharmacy of Craiova, Craiova, Romania

**Keywords:** emigration, mental health, residents, training

## Abstract

**Introduction:**

Important changes have been done in economic status of residents in 2018. The impact of these measures in changing opinion was checked.

**Objectives:**

The prioritization exercises of main factors related with psychiatric residents’ decision to emigrate could be a starting point of elaboration of a strategy of reforms.

**Methods:**

The study was cross sectional evaluation at national level on a randomized selected sample of Romanian psychiatric residents’ opinion on factors which influence decision of migration in EU countries in two time points: 2015 and 2020.

**Results:**

38% of residents intend to work abroad comparing with 78% before the economic changes (25.84% versus 71.66% for a limited period of time and 15.73% versus 28.33% intend to emigrate) and 2% versus 5% intend to leave the speciality. The most important factors for decision to emigrate changed from “Better working conditions” (15.73 versus 37.31% residents) to “Better training” 20.25% residents and the factor “respect and appreciation by colleagues” remained important for 19.10% versus 17.91%. “Lack of working place for partner” was considered by 26.96% of responders as an important disadvantage of working abroad. “Being far from family members” which was considered 5 years ago by 64.18% of responders as an important disadvantage of working abroad, nowadays concerns only 6.74%, probably because it seems easier to go abroad together with the family members.

**Conclusions:**

The factors (better training in psychiatry and psychotherapy, better supervision, more involvement in research) which influence the residents’ decision to emigrate represent the starting points on futures reforms in educational and medical system in psychiatry.

**Disclosure:**

No significant relationships.

